# Sample size requirements and intra-cluster correlations for stepped wedge cluster randomised trials in intensive care medicine: A practical guide

**DOI:** 10.1016/j.ccrj.2026.100168

**Published:** 2026-02-20

**Authors:** Thomas Hughes-Gooding, Diva Baggio, Edward Litton, David Pilcher, Paul J. Young, Jessica Kasza

**Affiliations:** a Intensive Care Unit, Wellington Hospital, Wellington, New Zealand; b School of Public Health and Preventive Medicine, Monash University, Melbourne, Victoria, Australia; c Australian and New Zealand Intensive Care Society Centre for Outcome and Resource Evaluation, Melbourne, Victoria, Australia; d Intensive Care Unit, Fiona Stanley Hospital, Murdoch, Western Australia, Australia; e Department of Intensive Care and Hyperbaric Medicine, The Alfred Hospital, Melbourne, Victoria, Australia; f Medical Research Institute of New Zealand, Wellington, New Zealand; g Australian and New Zealand Intensive Care Research Centre, Monash University, Melbourne, Victoria, Australia; h Department of Critical Care, University of Melbourne, Melbourne, Victoria, Australia

**Keywords:** Stepped wedge trial, Cluster randomised trial, Sample size determination, Intra-cluster correlation, Intensive care units

## Abstract

**Objective:**

To estimate key statistical parameters and provide practical guidance for planning stepped wedge cluster randomised trials in Australian and New Zealand intensive care units (ICUs).

**Design:**

Cross-sectional retrospective observational study using routinely collected ICU data.

**Setting:**

Adult public hospital ICUs contributing to the Australian and New Zealand Intensive Care Society Adult Patient Database between 2010 and 2023.

**Participants:**

All adult ICU admissions to 132 ICUs. Subgroups included unplanned admissions and admissions involving invasive mechanical ventilation or vasopressor use.

**Main outcome measures:**

In-hospital mortality during the index hospitalisation within 90 days of ICU admission. Intra-cluster correlation coefficients (ICCs) and cluster auto-correlations (CACs) were estimated using exchangeable, block-exchangeable, and discrete time decay models using a cross-sectional design.

**Results:**

Among 1,291,849 eligible ICU admissions, observed mortality ranged from 10.3% (all ICU admissions) to 23.0% (non-elective invasively ventilated patients in Mega-ROX ICUs). ICCs ranged from 0.008 to 0.022 and CACs from 0.83 to 1.00, with block-exchangeable or discrete time decay models most often providing the best fit. In a worked example, a 50-ICU stepped wedge trial with 10 steps (11 two-month periods) enrolling 45 unplanned ventilated patients per ICU per period (total ≈24,750 patients) would have 81.6% power to detect an absolute mortality reduction of 2.7%.

**Conclusions:**

Stepped wedge cluster randomised trials are feasible for evaluating ICU-wide interventions when routine data are available. The ICC and CAC estimates presented here provide Australian and New Zealand-specific parameters for future trial planning and demonstrate the potential of this design for pragmatic large-scale ICU research.

## 1 Introduction

The stepped wedge cluster randomised trial is a novel study design and one of several cluster randomised trial designs (alongside parallel group and cluster crossover).^1^ Stepped wedge cluster randomised trials can be used to evaluate ubiquitous interventions in intensive care unit (ICU) patients. In this design, each ICU represents a cluster and there is random and sequential crossover of ICUs from control to intervention until all ICUs are exposed to the study intervention.^2^ Any intervention that is effectively implemented ICU-wide to all patients, or to a subgroup of patients, may be evaluated using this design. In a stepped wedge trial, individual ICUs contribute patients under both control and intervention conditions at different time periods, which can improve efficiency when compared with a parallel group cluster trial. The design is most useful when a parallel group randomised trial would be underpowered, when the intervention cannot be easily withdrawn or reversed (making cluster crossover design impractical), or when a policy change is being implemented regardless of trial results.^3^

Despite the potential usefulness of stepped wedge cluster randomised trials such trials have rarely been conducted in Australian and New Zealand (ANZ) ICUs. To our knowledge, only one such trial evaluating a nurse-led delirium intervention has been reported from this region.^4^ In part, this may be because the sample size calculations are more complicated than for individual patient randomised trials and because data needed to conduct sample size calculations are not readily accessible. Sample size calculations for stepped wedge trials require specification of additional parameters compared to those required for an individually randomised trial, or even for a parallel group cluster randomised trial.

Our primary aim was therefore to estimate these parameters and to provide practical guidance on evaluating sample size requirements for stepped wedge cluster randomised trials. Our focus was on key populations of critically ill patients, and we used data from the Australian and New Zealand Intensive Care Society Adult Patient Database (ANZICS APD),^5^ with in-hospital mortality during the index hospitalisation within 90 days as the primary outcome. Our secondary aim was to illustrate the application of a stepped wedge cluster randomised trial design, using an online sample size calculator (https://clusterrcts.shinyapps.io/rshinyapp/)^6^ to provide a worked example of an envisaged future ICU-wide stepped wedge cluster randomised trial.

## 2 Methods

### 2.1 Study design

Retrospective observational study using deidentified data from the ANZICS APD. The ANZICS APD is a clinical quality registry dataset, managed by the ANZICS Centre for Outcome and Resource Evaluation. It contains data from over 200 ICUs describing more than 200000 admissions annually to adult ICUs representing 98% of ICUs in Australia and 67% of ICUs in New Zealand.^5^ With the exception of one hospital, each contributing ICU is the sole adult ICU in its hospital and provides all critical care with independent staffing and management.

Ethical approval for this study was obtained from the Alfred Hospital Ethics Committee (ref 134/25).

### 2.2 Setting and data source

We extracted data on all ICU admissions to public hospitals from January 2010 to December 2023 inclusive. These data were used to inform sample size calculations based on the entire patient cohort and also for the subset of ICUs participating in the Mega Randomised Oxygen (Mega-ROX) trial.^7^ We provided data for the Mega-ROX ICU subset as we are planning a stepped wedge trial of oxygen targets and reasoned that this group of ICUs would be the most likely to participate in such a trial. As the number of ICU admissions tends to increase with time, to avoid underestimation, we only used 2023 data to estimate the number of eligible patients per ICU per cluster.

### 2.3 Study population

All adult patients admitted to one of 132 adult-only or mixed adult-paediatric public ICUs within the study period were eligible for inclusion in this study. Where patients had more than one ICU admission between 2010 and 2023, only the first eligible admission was included in the analysis. Patients admitted for palliative care or to facilitate organ donation were excluded as such patients would usually not be included in a clinical trial.

### 2.4 Subgroups of interest

Data were further described for specific subgroups likely to represent common target ICU populations for future stepped wedge cluster trials. These subgroups were:

• Unplanned ICU admissions
• ICU admissions where patients received invasive mechanical ventilation (all admission episodes and unplanned admissions only were considered as separate subgroups of interest)
• ICU admissions where patients received vasopressors (all admission episodes and unplanned admissions only were considered as separate subgroups of interest)

Both overall, and for each subgroup, we provided data for all public hospital ICUs and for admissions to Mega-ROX ICUs separately.

### 2.5 Primary outcome

The primary outcome, used to inform sample size calculations, was in-hospital mortality during the index hospitalisation within 90 days of ICU admission.

### 2.6 Key parameters to determine the required sample size for a stepped wedge cluster trial

Key statistical parameters required to determine the sample size required for a stepped wedge cluster trial are described in Table 1.

**Table 1:** Key statistical parameters to determine the sample size for a stepped wedge cluster trial.

Parameter	Description	Relevance to sample size
Cluster size	The number of patients in the population of interest within each cluster (ICU) in each study period^a^.	Larger cluster sizes typically reduce the number of clusters needed to achieve statistical power, but there are diminishing returns as cluster sizes increase.
Cluster size coefficient of variation (CV)	A ratio describing the degree to which cluster sizes per ICU per study period vary relative to their average size across the entire study population.	A higher coefficient value indicates greater variability. More variation reduces efficiency and may require more clusters to maintain power.
Within-cluster correlation structure	Describes how the ICC and CAC combine for each pair of observations in a cluster and therefore the within-cluster correlation structure over time. There may be no change, a difference only between observations within the same period or only across periods, or decaying correlation as the time between observations increases.	If the correlation within clusters does not change over time, there is less variation within clusters across study periods and lower sample sizes are required. Assessing the sensitivity of sample size calculations to different assumptions about the within-cluster correlation structure is important.
Intra-cluster correlation (within-period ICC)	A measure of how similar two individuals in the same ICU are in the same study period, where a higher value indicates a greater degree of similarity.	A lower ICC means the effect of clustering is small and the design inflation to account for clustering is reduced (i.e. a smaller sample size is needed)
Cluster auto-correlation (CAC)	The CAC describes the correlation between two observations in the same cluster, measured one study period apart.	A higher CAC means outcomes in different study periods are more highly correlated, so each cluster provides less variable data and additional periods contribute less new information. A CAC of 1 indicates that there is no change over time.
Control event rate (CER)	The proportion of patients in the control group who experience the outcome of interest (i.e. in this case, in-hospital mortality)	Determines the baseline risk in the control group, which is essential when considering establishing the minimum effect size a study needs to detect.

ICU, intensive care unit.

a Study period: in stepped wedge designs, time is divided up into “study periods” of equal length.

### 2.7 Statistical methods

Estimates of intra-cluster correlations (ICCs) and cluster auto-correlations (CACs) were obtained from statistical models fit to the outcome of interest. To obtain estimates of the ICC and CAC on the same scale as the outcome, linear mixed models were fit to the outcome.^8^ Although the primary outcome is binary, linear mixed models provide correlation parameters directly on the scale of the observed outcome. In contrast, logistic regression models estimate correlations on the scale of a latent continuous variable that is dichotomised to give a binary outcome, so the resulting ICCs have a different interpretation and are not directly comparable for sample size calculations.

Three models, corresponding to different assumptions about the within-cluster correlation structure were fitted using a cross-sectional design:

• The exchangeable model: this model assumes that there is no change in the ICC across study periods (CAC = 1)^9^
• The block-exchangeable (aka two-period decay) model: this model allows the CAC to be less than 1, implying that individuals in the same ICU in the same study period have outcomes that are more highly correlated than individuals in the same ICU but in different study periods. However, the correlation between individuals in the same ICU but different study periods does not depend on the time between the study periods^10^
• The discrete time decay model: this model allows the CAC to be less than 1, and allows the correlation between two individuals in the same ICU but different study periods to decay the further apart in time their study periods are.^11^

Statistical notation for each model is provided in Table S1 in the Supplementary Appendix. Models include categorical effects for study periods and random effects for each ICU (and for each study period within ICU for the block-exchangeable and discrete time decay models). The ICC and CAC estimates for each model depend on the length of study periods and were calculated using data from 2020 to 2023 only, to approximate contemporary practice.^12^ The model that fits the data best is the model with the lowest Akaike information criterion.^13^ Models were fitted for the overall population and for restricting the analysis to specified subgroups. We made a pragmatic decision to focus on trial designs with two-month periods because we reasoned that such a period would both be realistic for implementation practicalities and balance stability in cluster size with overall study duration. However, we also considered alternative designs based on one-, three-, and six-month periods.

Although our focus is on stepped wedge cluster randomised trials, the ICC estimates are also directly applicable to other ICU cluster trial designs, including cluster crossover designs.

### 2.8 Software

This work was supported by Monash University through the Monash eResearch Centre and Helix, using the University-hosted Secure eResearch Platform (Monash SeRP) on the Nectar Research Cloud. The Nectar Research Cloud is a Collaborative Australian research platform supported by the National Collaborative Research Infrastructure Strategy. Analyses were conducted in R (version 4.5.0) and RStudio, using glmmTMB^14^ and lme4^15^ R packages.

### 2.9 Example sample size calculation

In the worked example, “sample size calculation” refers to calculating the minimum detectable difference for a prespecified trial design given the available number of clusters and patients.

To demonstrate an online stepped wedge cluster randomised trial sample size calculator (https://clusterrcts.shinyapps.io/rshinyapp/), we considered a trial with 10 sequences and 11 periods, each of two months’ duration, with five ICUs randomised to each sequence (50 ICUs in total; Supplementary Appendix). Cluster periods were defined by calendar-time admissions. Patients are assigned to the period in which they are admitted, and the 90-day in-hospital mortality outcome would then be attributed to that admission period. This example used the subgroup of non-elective ICU admissions requiring invasive mechanical ventilation at Mega-ROX ICUs.

### 2.10 Sample size estimates using adjusted models

For the study design used in the example sample size calculation, we evaluated two additional adjusted models: adjusting for the ICU-level variables of ICU type (metropolitan, rural/regional, tertiary) and jurisdiction (state or territory of Australia, or New Zealand); adjusting for those ICU-level variables as well as individual-level characteristics of age, sex, and Acute Physiology and Chronic Health Evaluation 2 score.

## 3 Results

### 3.1 Participants & ICUs

A total of 1,291,849 patients meeting study eligibility criteria were identified from the ANZICS APD, with admissions to 132 individual adult or mixed adult/paediatric ICUs.

### 3.2 Descriptive data

ICU-level characteristics are summarised in Table 2. The majority of ICUs were in Australia (113 of 132, 85.6%). A total of 39 (29.5%), 50 (37.9%), and 43 (32.6%) ICUs were classified as metropolitan, rural/regional, or tertiary respectively. Patient-level characteristics are summarised in Table S2 and Table S3 in the Supplementary Appendix. The median age of included patients was 63.7 years (interquartile range (IQR) 49.0–74.5), and a majority were male (58.4%). The median Acute Physiology and Chronic Health Evaluation-2 score at presentation was 15.0 (IQR 11.0–21.0). Mean numbers of patients in each population of interest per ICU per period length (one, two, three, or six months), with coefficients of variation, are provided in Table S4.

**Table 2:** ICU-level characteristics for all ICUs and for active trial sites.

Characteristic	All ICUs	Active trial sites
Total number of ICUs	n = 132	n = 56
Country, n (%)		
Australia	113 (85.6)	43 (76.8)
New Zealand	19 (14.4)	13 (23.2)
Jurisdiction, n (%)		
ACT	2 (1.5)	0 (0.0)
NSW	41 (31.1)	12 (21.4)
NT	2 (1.5)	2 (3.6)
NZ	19 (14.4)	13 (23.2)
QLD	21 (15.9)	8 (14.3)
SA	7 (5.3)	4 (7.1)
TAS	3 (2.3)	0 (0.0)
VIC	30 (22.7)	15 (26.8)
WA	7 (5.3)	2 (3.6)
Hospital classification, n (%)		
Metropolitan	39 (29.5)	16 (28.6)
Rural/regional	50 (37.9)	14 (25.0)
Tertiary	43 (32.6)	26 (46.4)

ACT: Australian Capital Territory; ICU: intensive care unit; NSW: New South Wales; NT: Northern Territory; NZ: New Zealand; QLD: Queensland; SA: South Australia; TAS: Tasmania; VIC: Victoria; WA: Western Australia.

### 3.3 Key statistical parameters and detectable effect sizes

Key statistical parameters to inform sample size calculations for populations of interest for all public hospital ICUs and for Mega-ROX ICUs, using two-month treatment periods, are shown in Table 3 and Table 4, respectively. Observed in-hospital mortality during the index hospitalisation within 90 days of ICU admission in all public hospital ICUs ranged from 10.3% in the *all ICU admissions* population to 22.4% in the *invasively ventilated during an unplanned admission* population. In each population, mortality rates were higher in Mega-ROX ICUs than in the broader all public hospital ICU cohort. In Mega-ROX ICUs, mortality rates ranged from 11.0% for the *all ICU admissions* population to 23.0% for the *invasively ventilated during an unplanned admission* population.

**Table 3:** Data for key statistical parameters required to calculate sample size for a stepped wedge cluster randomised clinical trial using data from all public hospital ICUs in Australia and New Zealand based on steps occurring every two months^a^.

Population of interest	Cluster size^b^	Cluster size coefficient of variation	Best model for within-cluster correlation structure^c^	ICC^d^	CAC^d^	Control event rate^e^
All ICU admissions	148 ± 113	0.76	Discrete time decay	0.011	0.99	10.3%
Non-elective ICU admissions	100 ± 63	0.63	Block-exchangeable	0.018	0.93	13.2%
Invasively ventilated	58 ± 65	1.13	Block-exchangeable	0.021	0.92	15.6%
Invasively ventilated during a non-elective admission	33 ± 31	0.93	Block-exchangeable	0.016	0.88	22.4%
Received vasopressors	75 ± 71	0.95	Block-exchangeable	0.012	0.94	15.3%
Received vasopressors during a non-elective ICU admission	49 ± 39	0.79	Block-exchangeable	0.012	0.99	20.1%

ANZICS APD: Australian and New Zealand Intensive Care Society Adult Patient Database; CI: confidence interval; ICC: Intra-cluster correlation; ICU: intensive care unit; SD: standard deviation.

a Unless otherwise specified, data were from 2023 ICU admission episodes recorded in the ANZICS APD. Patients admitted to the ICU for palliative care or to facilitate organ donation were excluded from all populations of interest.

b The cluster size is the mean ± SD number of patients in the population of interest admitted per ICU in a two month period.

c The model that fit the data best was the model determined to have the lowest Akaike information criterion.

d The ICC and CAC were calculated using the best-fitting model for 2 month periods based on data from 2020 to 2023.

e The control event rate was the observed mortality during the index hospitalisation within 90 days of ICU admission.

**Table 4:** Data for key statistical parameters required to calculate sample size for a stepped wedge cluster randomised clinical trial using data from Mega-ROX ICUs in Australia and New Zealand based on steps occurring every two months^a^.

Population of interest	Cluster size^b^	Cluster size coefficient of variation	Best model for within-cluster correlation structure^c^	ICC^d^	CAC^d^	Control event rate^e^
All ICU admissions	180 ± 118	0.66	Discrete time decay	0.009	0.990	11.0%
Non-elective ICU admissions	123 ± 66	0.54	Block-exchangeable	0.018	0.930	14.2%
Invasively ventilated	75 ± 69	0.93	Discrete time decay	0.022	0.990	16.0%
Invasively ventilated during a non-elective admission	45 ± 35	0.78	Block-exchangeable	0.018	0.880	23.0%
Received vasopressors	93 ± 72	0.78	Discrete time decay	0.012	0.990	15.7%
Received vasopressors during a non-elective ICU admission	63 ± 41	0.65	Discrete time decay	0.013	0.990	20.7%

ANZICS APD: Australian and New Zealand Intensive Care Society Adult Patient Database; CI: confidence interval; ICC: Intra-cluster correlation; ICU: intensive care unit; SD: standard deviation.

a Unless otherwise specified, data were from 2023 ICU admission episodes recorded in the ANZICS APD. Patients admitted to the ICU for palliative care or to facilitate organ donation were excluded from all populations of interest.

b The cluster size is the mean ± SD number of patients in the population of interest admitted per ICU in a two month period.

c The model that fit the data best was the model determined to have the lowest Akaike information criterion.

d The ICC and CAC were calculated using the best-fitting model for 2 month periods based on data from 2020 to 2023.

e The control event rate was the observed mortality during the index hospitalisation within 90 days of ICU admission.

Detectable effect sizes for stepped wedge cluster randomised clinical trials using a design with 50 ICUs where five ICUs are randomised to commence an intervention at a time every two months, giving 10 steps and 11 treatment periods, are shown in Table 5. Calculations consistently indicated that smaller treatment effects would be detectable in Mega-ROX ICUs than in the broader cohort of all public ANZ hospitals, in part reflecting a larger number of admissions in each treatment period in Mega-ROX ICUs.

**Table 5:** Detectable effect sizes for stepped wedge cluster randomised clinical trials using a design with 50 ICUs where five ICUs are randomised to commence an intervention at a time every two months giving 10 steps and 11 treatment periods^a^.

Population of interest	Control event rate^b^	Correlation structure used for calculations^c^	Effect-size detectable with 80% power^d^		Effect-size detectable with 90% power^d^		Total number of participants that would be included
			Absolute risk reduction	Relative risk reduction	Absolute risk reduction	Relative risk reduction	
All ICUs							
All ICU admissions	10.3%	Discrete time decay	1.1 percentage points	10.7%	1.2 percentage points	11.2%	81,400
Non-elective ICU admissions	13.2%	Block-exchangeable	1.5 percentage points	11.4 %	1.7 percentage points	12.9%	55,000
Invasively ventilated	15.6%	Block-exchangeable	2.1 percentage points	13.5%	2.4 percentage points	15.4%	31,900
Invasively ventilated during a non-elective admission	22.4%	Block-exchangeable	3.0 percentage points	13.4%	3.5 percentage points	15.6%	18,150
Received vasopressors	15.3%	Block-exchangeable	1.8 percentage points	11.8%	2.0 percentage points	13.1%	41,250
Received vasopressors during a non-elective ICU admission	20.1%	Block-exchangeable	2.3 percentage points	11.4%	2.6 percentage points	12.9%	26,950
Mega-ROX ICUs							
All ICU admissions	11.0%	Discrete time decay	1.0 percentage points	9.1%	1.2 percentage points	10.9%	99,000
Non-elective ICU admissions	14.2%	Block-exchangeable	1.4 percentage points	9.9%	1.6 percentage points	11.2%	67,650
Invasively ventilated	16.0%	Discrete time decay	1.8 percentage points	11.3%	2.0 percentage points	12.5%	41,250
Invasively ventilated during a non-elective admission	23.0%	Block-exchangeable	2.7 percentage points	11.7%	3.1 percentage points	13.5%	24,750
Received vasopressors	15.7%	Discrete time decay	1.6 percentage points	10.2%	1.8 percentage points	11.9%	51,150
Received vasopressors during a non-elective ICU admission	20.7%	Discrete time decay	2.1 percentage points	10.1%	2.4 percentage points	11.6%	34,650

ANZICS APD: Australian and New Zealand Intensive Care Society Adult Patient Database; CI: confidence interval; ICC: Intra-cluster correlation; ICU: intensive care unit; SD: standard deviation.

a Unless otherwise specified, data were from 2023 ICU admission episodes recorded in the ANZICS APD. Patients admitted to the ICU for palliative care or to facilitate organ donation were excluded from all populations of interest.

b The control event rate was the observed mortality during the index hospitalisation within 90 days of ICU admission.

c The model that fit the data best was the model determined to have the lowest Akaike information criterion. When the block-exchangeable model had the best fit, varying cluster sizes were accounted for in the sample size calculation.

d Effect size calculations used the ICC and CAC as calculated using the best-fitting model for 2 month periods based on data from 2020 to 2023.

Detailed ICC and CAC estimates for all subgroups, period lengths (one, two, three, and six months) and within-cluster correlation structures are shown in Table S5–S8 in the Supplementary appendix.

### 3.4 Example sample size calculation

The detailed worked sample size calculation example for a population of patients receiving unplanned mechanical ventilation in Mega-ROX ICUs is shown in the Supplementary appendix. This example is based on a 50-ICU stepped wedge trial with 10 steps (11 two-month treatment periods) and would be expected to enrol a mean of 45 patients per ICU per treatment period, corresponding to a total sample size of 24,750 patients. Such a trial would provide 81.6% power to detect an absolute risk reduction of 2.7 percentage points. As ICU admissions tend to increase over time, this estimate may be conservative. For example, if the mean cluster size were to increase to 62, power of 90.1% would be expected. Values for ICC and CAC adjusting for specified ICU-level variables of ICU type (metropolitan, rural/regional, tertiary) and jurisdiction (state or territory of Australia, or New Zealand) with or without additional adjustment for specified individual-level characteristics, are shown in Table S9.

## 4 Discussion

In this retrospective study, we showed that for a hypothetical stepped wedge trial conducted across 50 ICUs, with five ICUs crossing over every two months (total recruitment 22 months), mortality effect sizes of 1.2–3.5 percentage points would be detectable with 90% power. The specific detectable effect depends on the target population and whether the trial involves all ANZ ICUs or only Mega-ROX ICUs. These statistical parameters can guide sample size estimation for stepped wedge ICU trials, though the large sample sizes required across all populations indicate substantial logistical challenges.

The stepped wedge cluster randomised trial is a complex design that is used increasingly in the evaluation of service-delivery interventions.^1^ It is particularly suited to situations where phased implementation is required due to logistical or resource constraints, and where minimising contamination is essential.^1^^,^^16^ However, other cluster randomised trial designs (such as parallel group or crossover) may offer greater simplicity and efficiency if carry-over of an intervention is unlikely. Stepped wedge designs are also particularly sensitive to secular and seasonal trends in outcomes (which are well recognised for ICU admission rates/mortality) and therefore require careful modelling of time effects.

By randomising the timing of intervention rollout across individual ICUs, all ICUs involved in the trial will eventually transition to the intervention, which can facilitate stakeholder engagement and align with real-world practice changes.^17^^,^^18^ This is particularly suited to system-level interventions (oxygen targets, sedation protocols, and delirium-prevention strategies) where an outcome (e.g. mortality) is routinely collected.^19^ Because of the large number of participants required to detect potentially plausible mortality effects,^20^ it is probable that a stepped wedge cluster trial would need to use readily available routine clinical information to be feasible. Our data suggest a stepped wedge trial design would not be feasible for complex interventions that require trial-specific collection of detailed data.

Hussey and Hughes established the importance of accounting for clustering and time effects in stepped wedge trial calculations,^9^ typically assuming a constant (exchangeable) correlation structure between periods. Subsequent methodological advances have highlighted the limitations of this assumption and introduced more flexible models allowing between-period correlation to differ from within-period correlation and to decay with increasing time separation.^10^^,^^11^^,^^21^ Our findings show that discrete time decay or block-exchangeable models most often best fit ICU data, and block-exchangeable models provide the most conservative estimates. Sensitivity analyses including transparent reporting of the chosen correlation structure and its justification are recommended.^22^^,^^23^ However, it is notable that uncertainties about the magnitude of expected treatment effects may be substantially larger than uncertainties related to model selection.^20^

The CLustered Outcome Dataset (CLOUD) bank aggregates ICC and CAC estimates from a range of cluster trials. Median within-period ICCs for these nonspecific settings are typically 0.02–0.09 and CACs (discrete time decay) around 0.19–0.91, but with substantial variation by period length and cluster size.^22^ Our reported ICC values range from 0.008 to 0.022 (depending on subgroup) and CAC values 0.83–1.00, demonstrating *less variation* between ICUs within a period and less variation *over time* within the same ICU with respect to our primary outcome when compared to aggregated data. Empirical estimates of ICCs in ICUs are sparse. A systematic review reported a median ICC of 0.047 (IQR 0.01–0.13) in critical care cluster-randomised trials, though only 12 of 59 (20%) of studies report ICCs at all.^24^

Prior data have shown within-period ICCs decrease with longer period lengths, and CAC values move closer to 1 with increasing cluster size.^22^^,^^25^ In our data, ICC and CAC vary minimally across period lengths, so step length can be chosen for logistics, with power driven primarily by the number of clusters and correlation structure chosen rather than period length.

Although stepped wedge trials are less sensitive to unequal cluster sizes than parallel group cluster randomised trials, cluster size variation is expected in ICU trials and high coefficients of variation can reduce power. Researchers recommend analysis adjustments for unequal cluster sizes for highly variable settings,^26^ and our data quantify the observed coefficient of variation and demonstrate power under alternative correlation structures to reflect uncertainty. The smaller detectable treatment effects in Mega-ROX ICUs likely reflect lower inter-cluster variability and larger average cluster sizes in these ICUs, and illustrate a trade-off between efficiency and generalisability; recruiting a smaller, more homogenous group of ICUs can increase power, whereas including a more comprehensive sample of ICUs may improve validity but requires a larger sample size and the inclusion of centres with more variable levels of research activity.

Our study has some strengths. We provide period-specific ICC and CAC estimates for a broad range of subgroups within the population of ANZ ICU admissions and then apply them directly to sample-size planning for a hypothetical large-scale trial in a commonly studied population of critically ill patients. By reporting values across multiple period lengths and clinically relevant subgroups, and through a step-by-step worked example, we hope to enable researchers to use the required correlation values and Shiny Cluster Randomised Trial (CRT) Calculator in planning future stepped wedge trials. As a result, this paper reduces key barriers that have previously limited stepped wedge trial adoption for ICU-wide interventions. We evaluated several plausible within-cluster correlation structures and demonstrated that model choice has implications for detectable effects.

We also present ICC and CAC estimates adjusted for ICU-level and individual-level characteristics, accounting for pre-existing systematic differences to present a re-estimated correlation value. For 2-month periods, ICC and CAC estimates were similar in unadjusted and ICU-level adjusted models, and were only modestly decreased when considering individual-level characteristics. This suggests the unadjusted models used in the analysis are robust and any subsequent sample size calculations are conservative.

We acknowledge certain limitations. Our correlation estimates are derived from the ANZICS APD data 2020–2023 inclusive for public hospital ICUs, and therefore the applicability of these estimates for private ICUs, non-ANZ ICUs, and for research in the future is uncertain. Nonetheless, the step-by-step process of sample size calculation using cross-sectional real-world data is generalisable to future ICU trial design. Although values are provided for different period lengths, since, by design, stepped wedge trials change practice ICU-wide at each step, the rate at which correlation decays between periods may not be consistent with that observed in routine care. There is a potential risk of interference between clusters (cross-site contamination), and case-mix within ICUs may vary significantly between periods beyond that accounted for in models due to unexpected external factors. External validation in other trials and with other geographical regions would help to improve knowledge gaps and enhance the feasibility of using a stepped wedge trial design in the ICU. The worked example relies on the publicly available Shiny CRT calculator. Although not explicitly an issue for our specific worked example, the calculator cannot simultaneously model discrete time decay and variable cluster size. The calculator also requires visual interpretation to identify the smallest number of clusters per sequence to achieve a target power across different ICC/CAC estimates, and does not directly present the power for a specified effect size at a fixed sample size. Therefore, a “trial and error” approach of differing step numbers may be required; since, by definition, cluster size, cluster size variation and other parameters are fixed/precalculated. Finally, although we use 2023 data to derive cluster size and outcomes, mortality trends and population demographics will continue to evolve. Any future proposed trial should use the most recent data available to minimise the risk of underpowering a trial or making it larger and more costly than necessary.

## 5 Conclusion

Stepped-wedge cluster randomised trials are a practical, useful, and pragmatic design for ICU-wide interventions in specific circumstances. Our work provides period-specific ICCs and CACs for one, two, three, and six-month periods in 132 ANZ ICUs to align with and extend a growing body of methodological and empirical research. We also demonstrate the practicalities of trial design using a publicly available calculator. With 45 unplanned ventilated admissions per ICU per 2-month period at Mega-ROX ICUs, a 50-ICU stepped wedge cluster randomised trial has 81.6% power to detect a 2.7 percent absolute risk reduction in 90-day mortality under conservative correlation assumptions.

## CRediT authorship contribution statement

**Hughes-Gooding:** Writing – Original Draft preparation **Baggio and Kasza:** Statistical analyses, Writing – Reviewing and Editing; **Litton and Pilcher:** Writing – Reviewing and Editing. **Young:** Conceptualisation, Writing – Reviewing and Editing.

## Funding

Paul Young, David Pilcher, and Ed Litton declare they are members of the Editorial Board for Critical Care and Resuscitation. This research was conducted during the tenure of a Health Research Council of New Zealand Clinical Practitioner Fellowship held by Paul Young. The Medical Research Institute of New Zealand is supported by Independent Research Organisation funding from the Health Research Council of New Zealand. Jessica Kasza is supported by an NHMRC Investigator Grant, ID 2033380.

## Declaration of competing interest

The authors declare the following financial interests/personal relationships which may be considered as potential competing interests: Jessica Kasza reports financial support was provided by the National Health and Medical Research Council. Paul Young reports financial support was provided by the Health Research Council of New Zealand. Given their role on the Editorial Board for Critical Care and Resuscitation, the following authors had no involvement in the peer review of this article and had no access to information regarding its peer review. Full responsibility for the editorial process for this article was delegated to another journal editor. Paul Young, David Pilcher, and Ed Litton. If there are other authors, they declare that they have no known competing financial interests or personal relationships that could have appeared to influence the work reported in this paper.
