# Supplementary material for: Sample size requirements and intra-cluster correlations for stepped wedge cluster randomised trials in intensive care medicine: A practical guide
Source: Crit Care Resusc. 2026 Feb 20;28(1):100168. doi: 10.1016/j.ccrj.2026.100168 (PMC12936732; doi:10.1016/j.ccrj.2026.100168)
Supplement: Multimedia component 1 [file mmc1.docx]

**SUPPLEMENTARY APPENDIX**

**Supplementary methods**

**Contributing ICUs**

The investigators acknowledge contribution of the staff who collected data that were used in this analysis. The contributing ICUs were: Albury Wodonga Health ICU; Alfred Hospital ICU; Alice Springs Hospital ICU; Armadale Health Service ICU; Ashford Community Hospital ICU; Auckland City Hospital CV ICU; Auckland City Hospital DCCM; Austin Hospital ICU; Ballarat Health Services ICU; Bankstown-Lidcombe Hospital ICU; Bathurst Base Hospital ICU; Bendigo Health Care Group ICU; Blacktown Hospital ICU; Bowral Hospital HDU; Box Hill Hospital ICU; Broken Hill Base Hospital & Health Services ICU; Bunbury Regional Hospital ICU; Bundaberg Base Hospital ICU; Caboolture Hospital ICU; Cairns Hospital ICU; Campbelltown Hospital ICU; Canberra Hospital ICU; Casey Hospital ICU; Central Gippsland Health Service (Sale) ICU; Christchurch Hospital ICU; Coffs Harbour Health Campus ICU; Concord Hospital (Sydney) ICU; Dandenong Hospital ICU; Dubbo Base Hospital ICU; Dunedin Hospital ICU; Echuca Regional Hospital HDU; Fairfield Hospital ICU; Fiona Stanley Hospital ICU; Flinders Medical Centre ICU;

Footscray Hospital ICU; Frankston Hospital ICU; Fremantle Hospital ICU; Gold Coast University Hospital ICU; Gosford Hospital ICU; Goulburn Base Hospital ICU;

Goulburn Valley Health ICU; Grafton Base Hospital ICU; Grampians Health Horsham ICU; Griffith Base Hospital ICU; Hawkes Bay Hospital ICU; Hervey Bay Hospital ICU; Hornsby Ku-ring-gai Hospital ICU; Hutt Hospital ICU; Ipswich Hospital ICU; John Hunter Hospital ICU; Joondalup Health Campus ICU; Latrobe Regional Hospital ICU;

Launceston General Hospital ICU; Lismore Base Hospital ICU; Liverpool Hospital ICU; Logan Hospital ICU; Lyell McEwin Hospital ICU; Mackay Base Hospital ICU; Maitland Hospital ICU; Maitland Private Hospital ICU; Manly Hospital & Community Health ICU; Manning Rural Referral Hospital ICU; Maroondah Hospital ICU; Mater Adults Hospital (Brisbane) ICU; Middlemore Hospital ICU; Mildura Base Public Hospital ICU; Modbury Public Hospital ICU; Monash Medical Centre-Clayton Campus ICU; Mount Hospital ICU; Mount Isa Hospital ICU; Nambour General Hospital ICU; Nelson Hospital ICU; Nepean Hospital ICU; Noosa Hospital ICU; North Canberra Hospital ICU; North Shore Hospital ICU; North West Regional Hospital (Burnie) ICU; Northeast Health Wangaratta ICU; Northern Beaches Hospital ICU

Orange Base Hospital ICU; Peter MacCallum Cancer Institute ICU; Port Macquarie Base Hospital ICU; Prince of Wales Hospital (Sydney) ICU; Princess Alexandra Hospital ICU; Queen Elizabeth II Jubilee Hospital ICU; Redcliffe Hospital ICU;

Repatriation General Hospital (Adelaide) ICU; Robina Hospital ICU; Rockhampton Hospital ICU; Rockingham General Hospital ICU;Rotorua Hospital ICU; Royal Adelaide Hospital ICU; Royal Brisbane and Women's Hospital ICU; Royal Darwin Hospital ICU; Royal Hobart Hospital ICU; Royal Melbourne Hospital ICU; Royal North Shore Hospital ICU; Royal Perth Hospital ICU; Royal Prince Alfred Hospital ICU; Ryde Hospital and Community Health Services ICU; Shoalhaven Hospital ICU;

Sir Charles Gairdner Hospital ICU; South East Regional Hospital ICU; South West Healthcare (Warrnambool) ICU; St Andrew's Hospital (Adelaide) ICU; St Andrew's Hospital Toowoomba ICU; St Andrew's War Memorial Hospital ICU; St George Hospital (Sydney) CICU; St George Hospital (Sydney) ICU; St George Hospital (Sydney) ICU2; St Vincent's Hospital (Melbourne) ICU; St Vincent's Hospital (Sydney) ICU; St Vincent's Hospital (Toowoomba) ICU; St Vincent's Private Hospital (Sydney) ICU; St Vincent's Private Hospital Fitzroy ICU; Sunnybank Hospital ICU

Sunshine Coast University Hospital ICU; Sunshine Coast University Private Hospital ICU; Sunshine Hospital ICU; Sutherland Hospital & Community Health Services ICU;

Sydney Adventist Hospital ICU; Tamworth Base Hospital ICU; Taranaki Health ICU; Tauranga Hospital ICU; The Bays Hospital ICU; The Chris O’Brien Lifehouse ICU; The Memorial Hospital (Adelaide) ICU; The Northern Hospital ICU; The Prince Charles Hospital ICU; The Queen Elizabeth (Adelaide) ICU; The Wesley Hospital ICU; Timaru Hospital ICU; Toowoomba Hospital ICU; Townsville University Hospital ICU; Tweed Heads District Hospital ICU; University Hospital Geelong ICU; Wagga Wagga Base Hospital & District Health ICU; Waikato Hospital ICU; Wairau Hospital ICU; Wellington Hospital ICU; Werribee Mercy Hospital ICU; Western District Health Service (Hamilton) ICU; Western Hospital (SA) ICU; Westmead Hospital ICU; Whakatane Hospital ICU; Whangarei Area Hospital - Northland Health Ltd ICU; Wollongong Hospital ICU; Wyong Hospital ICU

| **Table S1:** Linear mixed effects models and definitions of correlation parameters for various models: Adapted from Ouyang et al., 2023^26^ | | | | |
| --- | --- | --- | --- | --- |
| Correlation structure | Model equation  $i=1,\ldots, K$ denotes cluster  $j=1,\ldots, T$ denotes period  $k=1,\ldots,m$denotes individual | Within-period ICC (ICC) | Between-period ICC ($ICC_{BP}$) | CAC  $(\frac{ICC_{BP}}{ICC})$ |
| Exchangeable | $Y_{ijk}=\mu+\beta_{j}+\alpha_{i}+ \varepsilon_{ijk}$  $\sum\beta_{j}=0$  $\alpha_{i}\sim N(0, \sigma_{\alpha}^{2})$  $\varepsilon_{ijk}\sim N(0, \sigma_{\varepsilon}^{2})$ | $\frac{\sigma_{\alpha}^{2}}{\sigma_{\alpha}^{2}+\sigma_{\varepsilon}^{2}}$ | $\frac{\sigma_{\alpha}^{2}}{\sigma_{\alpha}^{2}+\sigma_{\varepsilon}^{2}}$ | $1$ |
| Block exchangeable | $Y_{ijk}=\mu+\beta_{j}+\alpha_{i}+\gamma_{ij}+ \varepsilon_{ijk}$  $\sum\beta_{j}=0$  $\alpha_{i}\sim N(0, \sigma_{\alpha}^{2})$  $\varepsilon_{ijk}\sim N(0, \sigma_{\varepsilon}^{2})$  $\gamma_{ij}\sim N(0, \sigma_{\gamma}^{2})$ | $\frac{\sigma_{\alpha}^{2}+\sigma_{\gamma}^{2}}{\sigma_{\alpha}^{2}+\sigma_{\gamma}^{2}+\sigma_{\varepsilon}^{2}}$ | $\frac{\sigma_{\alpha}^{2}}{\sigma_{\alpha}^{2}+\sigma_{\gamma}^{2}+\sigma_{\varepsilon}^{2}}$ | $\frac{\sigma_{\alpha}^{2}}{\sigma_{\alpha}^{2}+\sigma_{\gamma}^{2}}$ |
| Discrete time decay | $Y_{ijk}=\mu+\beta_{j}+\alpha_{i}+\gamma_{ij}+ \varepsilon_{ijk}$  $\sum\beta_{j}=0$  $\alpha_{i}\sim N(0, \sigma_{\alpha}^{2})$  $\varepsilon_{ijk}\sim N(0, \sigma_{\varepsilon}^{2})$  $\boldsymbol{\gamma}_{i}=\left( \gamma_{i1}, \ldots, \gamma_{iT} \right)^{T}\sim N\left( 0, \boldsymbol{\Sigma} \right)$; $\boldsymbol{\Sigma}$ is a matrix with entry (s,t) denoting the covariance between $\gamma_{is}$ and $\gamma_{it}$: ${cov(\gamma}_{is}, \gamma_{it})=\sigma_{\gamma}^{2} r^{\vert s-t\vert}$ | $\frac{\sigma_{\gamma}^{2}}{\sigma_{\gamma}^{2}+\sigma_{\varepsilon}^{2}}$ | $\frac{\sigma_{\gamma}^{2}}{\sigma_{\gamma}^{2}+\sigma_{\varepsilon}^{2}}\cdot r^{\vert s-t\vert}$  For the ICC between periods $s$ and $t$ | $r$ |

To allow for interpretability of $\mu$ as the mean outcome across all time periods, the sum of scalars $\beta_{j}$ of time effects is subjected to the constraint $\sum\beta_{j}=$

### **Supplementary Results**

| **Table S2:** Patient-level characteristics for the whole population and pre-specified subgroups. | | | | | | | |
| --- | --- | --- | --- | --- | --- | --- | --- |
| Characteristic | Total population | Unplanned admissions | Ventilated patients (total) | Ventilated patients (unplanned admissions only) | Vasopressors (total) | Vasopressors (unplanned admissions only) | Patients in active trial sites |
| Total number of patients | n=1291849 | n=442199 | n=244924 | n=144577 | n=255546 | n=165925 | n=709426 |
| Number of ICUs | n=132 | n=125 | n=124 | n=124 | n=122 | n=122 | n=56 |
| Sex, n (%) |  |  |  |  |  |  |  |
| Female | 537569 (41.6) | 194207 (43.9) | 85760 (35.0) | 55610 (38.5) | 97951 (38.3) | 69220 (41.7) | 285954 (40.3) |
| Intersex | 312 (0.0) | 236 (0.1) | 106 (0.0) | 74 (0.1) | 123 (0.0) | 88 (0.1) | 139 (0.0) |
| Male | 753819 (58.4) | 247690 (56.0) | 159012 (64.9) | 88863 (61.5) | 157428 (61.6) | 96589 (58.2) | 423238 (59.7) |
| Missing | 149 (0.0) | 66 (0.0) | 46 (0.0) | 30 (0.0) | 44 (0.0) | 28 (0.0) | 95 (0.0) |
| Country, n (%) |  |  |  |  |  |  |  |
| Australia | 1135887 (87.9) | 393823 (89.1) | 213361 (87.1) | 126817 (87.7) | 218048 (85.3) | 143471 (86.5) | 594021 (83.7) |
| New Zealand | 155962 (12.1) | 48376 (10.9) | 31563 (12.9) | 17760 (12.3) | 37498 (14.7) | 22454 (13.5) | 115405 (16.3) |
| Jurisdiction, n (%) | |  |  |  |  |  |  |
| ACT | 29919 (2.3) | 10377 (2.3) | 5187 (2.1) | 3436 (2.4) | 6787 (2.7) | 4729 (2.9) | 0 (0.0) |
| NSW | 395579 (30.6) | 140625 (31.8) | 62927 (25.7) | 36149 (25.0) | 73027 (28.6) | 47669 (28.7) | 181210 (25.5) |
| NT | 15629 (1.2) | 6255 (1.4) | 1714 (0.7) | 1587 (1.1) | 2578 (1.0) | 2368 (1.4) | 15629 (2.2) |
| NZ | 155962 (12.1) | 48376 (10.9) | 31563 (12.9) | 17760 (12.3) | 37498 (14.7) | 22454 (13.5) | 115405 (16.3) |
| QLD | 206759 (16.0) | 61201 (13.8) | 47169 (19.3) | 26939 (18.6) | 46054 (18.0) | 28333 (17.1) | 84073 (11.9) |
| SA | 85635 (6.6) | 30434 (6.9) | 17687 (7.2) | 10628 (7.4) | 9011 (3.5) | 7240 (4.4) | 84246 (11.9) |
| TAS | 25104 (1.9) | 7058 (1.6) | 3152 (1.3) | 2047 (1.4) | 2627 (1.0) | 1610 (1.0) | 0 (0.0) |
| VIC | 300021 (23.2) | 107335 (24.3) | 54868 (22.4) | 33544 (23.2) | 57977 (22.7) | 39340 (23.7) | 205873 (29.0) |
| WA | 77241 (6.0) | 30538 (6.9) | 20657 (8.4) | 12487 (8.6) | 19987 (7.8) | 12182 (7.3) | 22990 (3.2) |
| Died within 90 days of ICU admission, n (%) | 129322 (10.0) | 57103 (12.9) | 37485 (15.3) | 31487 (21.8) | 40089 (15.7) | 34576 (20.8) | 77148 (10.9) |
| Median age, years (IQR) | 63.7  (49.0 to 74.5) | 63.0  (46.6 to 74.8) | 61.1  (46.7 to 71.7) | 57.7  (41.0 to 70.7) | 65.6  (53.4 to 74.9) | 65.3  (51.5 to 75.5) | 63.1  (48.4 to 74.0) |
| Median APACHE-2 Score (IQR) | 15.0  (11.0 to 21.0) | 16.0  (11.0 to 22.0) | 18.0  (13.0 to 24.0) | 20.0  (14.0 to 27.0) | 18.0  (14.0 to 24.0) | 20.0  (15.0 to 26.0) | 15.0  (11.0 to 21.0) |
| Abbreviations: APACHE-2: Acute Physiology and Chronic Health Evaluation 2; ACT: Australian Capital Territory; NSW: New South Wales; NT: Northern Territory; NZ: New Zealand; QLD: Queensland; SA: South Australia; TAS: Tasmania; VIC: Victoria; WA: Western Australia | | | | | | | |

| **Table S3:** Patient-level characteristics for patients in Mega-ROX ICUs and pre-specified subgroups. | | | | | | |
| --- | --- | --- | --- | --- | --- | --- |
| Characteristic | All patients in Mega-ROX ICUs | Unplanned admissions | Ventilated patients (total) | Ventilated patients (unplanned admissions only) | Vasopressors (total) | Vasopressors (unplanned admissions only) |
| Total number of patients | n=709426 | n=251221 | n=152315 | n=92732 | n=152205 | n=102148 |
| Number of ICUs | n=56 | n=56 | n=56 | n=56 | n=55 | n=55 |
| Sex, n (%) |  |  |  |  |  |  |
| Female | 285954 (40.3) | 107164 (42.7) | 52121 (34.2) | 34634 (37.3) | 57167 (37.6) | 41624 (40.7) |
| Intersex | 139 (0.0) | 103 (0.0) | 49 (0.0) | 30 (0.0) | 58 (0.0) | 41 (0.0) |
| Male | 423238 (59.7) | 143912 (57.3) | 100109 (65.7) | 58044 (62.6) | 94947 (62.4) | 60462 (59.2) |
| Missing | 95 (0.0) | 42 (0.0) | 36 (0.0) | 24 (0.0) | 33 (0.0) | 21 (0.0) |
| Country, n (%) |  |  |  |  |  |  |
| Australia | 594021 (83.7) | 209798 (83.5) | 128215 (84.2) | 76735 (82.7) | 123554 (81.2) | 82622 (80.9) |
| New Zealand | 115405 (16.3) | 41423 (16.5) | 24100 (15.8) | 15997 (17.3) | 28651 (18.8) | 19526 (19.1) |
| Jurisdiction, n (%) |  |  |  |  |  |  |
| ACT | 0 (0.0) | 0 (0.0) | 0 (0.0) | 0 (0.0) | 0 (0.0) | 0 (0.0) |
| NSW | 181210 (25.5) | 65023 (25.9) | 36265 (23.8) | 20871 (22.5) | 39568 (26.0) | 25512 (25.0) |
| NT | 15629 (2.2) | 6255 (2.5) | 1714 (1.1) | 1587 (1.7) | 2578 (1.7) | 2368 (2.3) |
| NZ | 115405 (16.3) | 41423 (16.5) | 24100 (15.8) | 15997 (17.3) | 28651 (18.8) | 19526 (19.1) |
| QLD | 84073 (11.9) | 27247 (10.8) | 22591 (14.8) | 13833 (14.9) | 21773 (14.3) | 14097 (13.8) |
| SA | 84246 (11.9) | 30344 (12.1) | 17664 (11.6) | 10605 (11.4) | 8993 (5.9) | 7222 (7.1) |
| TAS | 0 (0.0) | 0 (0.0) | 0 (0.0) | 0 (0.0) | 0 (0.0) | 0 (0.0) |
| VIC | 205873 (29.0) | 70843 (28.2) | 44871 (29.5) | 25123 (27.1) | 46254 (30.4) | 29457 (28.8) |
| WA | 22990 (3.2) | 10086 (4.0) | 5110 (3.4) | 4716 (5.1) | 4388 (2.9) | 3966 (3.9) |
| Died within 90 days of ICU admission,  n (%) | 77148 (10.9) | 35055 (14.0) | 23999 (15.8) | 20659 (22.3) | 24697 (16.2) | 21821 (21.4) |
| Median age, years (IQR) | 63.1 (48.4 to 74.0) | 62.2 (46.0 to 74.1) | 61.0 (46.4 to 71.5) | 57.5 (40.7 to 70.4) | 65.1 (52.8 to 74.4) | 64.5 (50.8 to 74.9) |
| Median APACHE-2 Score (IQR) | 15.0 (11.0 to 21.0) | 17.0 (12.0 to 23.0) | 18.0 (13.0 to 24.0) | 20.0 (14.0 to 26.0) | 18.0 (14.0 to 24.0) | 20.0 (15.0 to 26.0) |

Abbreviations: APACHE-2: Acute Physiology and Chronic Health Evaluation 2; ACT: Australian Capital Territory; NSW: New South Wales; NT: Northern

Territory; NZ: New Zealand; QLD: Queensland; SA: South Australia; TAS: Tasmania; VIC: Victoria; WA: Western Australia.

| **Table S4:** Means, standard deviations, and coefficients of variation of the number of patients for all ICUs, and Mega-ROX ICUs, per cluster, in each period (of length 1 month, 2 months, 3 months, 6 months) using 2023 data only. | | | | | |
| --- | --- | --- | --- | --- | --- |
| Period length (months) | Subgroup | Mean cluster size for all ICUs (SD)* | Coefficients of variation for admissions to all ICUs | Mean cluster size for Mega-ROX ICUs (SD)^†^ | Coefficients of variation for admissions to Mega-ROX ICUs |
| One | All admissions | 75 (56) | 0.75 | 90 (59) | 0.66 |
| One | Non-elective ICU admissions | 50 (32) | 0.63 | 62 (34) | 0.54 |
| One | Invasively ventilated | 29 (33) | 1.13 | 38 (35) | 0.93 |
| One | Invasively ventilated during a non-elective admission | 17 (16) | 0.95 | 23 (18) | 0.80 |
| One | Received vasopressors | 38 (36) | 0.95 | 47 (37) | 0.78 |
| One | Received vasopressors during a non-elective ICU admission | 25 (20) | 0.79 | 32 (21) | 0.66 |
| Two | All admissions | 148 (113) | 0.76 | 180 (118) | 0.66 |
| Two | Non-elective ICU admissions | 100 (63) | 0.63 | 123 (66) | 0.54 |
| Two | Invasively ventilated | 58 (65) | 1.13 | 75 (69) | 0.93 |
| Two | Invasively ventilated during a non-elective admission | 33 (31) | 0.93 | 45 (35) | 0.78 |
| Two | Received vasopressors | 75 (71) | 0.95 | 93 (72) | 0.78 |
| Two | Received vasopressors during a non-elective ICU admission | 49 (39) | 0.79 | 63 (41) | 0.65 |
| Three | All admissions | 222 (169) | 0.76 | 269 (176) | 0.66 |
| Three | Non-elective ICU admissions | 149 (93) | 0.63 | 185 (99) | 0.54 |
| Three | Invasively ventilated | 86 (97) | 1.13 | 111 (103) | 0.93 |
| Three | Invasively ventilated during a non-elective admission | 50 (46) | 0.93 | 67 (52) | 0.77 |
| Three | Received vasopressors | 113 (107) | 0.95 | 138 (108) | 0.78 |
| Three | Received vasopressors during a non-elective ICU admission | 74 (58) | 0.79 | 94 (61) | 0.65 |
| Six | All admissions | 445 (337) | 0.76 | 538 (352) | 0.66 |
| Six | Non-elective ICU admissions | 299 (186) | 0.62 | 370 (198) | 0.53 |
| Six | Invasively ventilated | 173 (194) | 1.12 | 223 (207) | 0.93 |
| Six | Invasively ventilated during a non-elective admission | 100 (92) | 0.92 | 135 (104) | 0.77 |
| Six | Received vasopressors | 225 (213) | 0.95 | 277 (215) | 0.78 |
| Six | Received vasopressors during a non-elective ICU admission | 148 (116) | 0.78 | 187 (121) | 0.65 |

*Representing the mean (SD) number of admissions per ICU, for each given period length and subgroup

†Representing the mean (SD) number of admissions per ICU enrolled in the Mega-ROX trial, for each given period length and subgroup

| **Table S5:** ICCs and CACs for specific subgroups for each of the three within-cluster correlation structures, period length one month | | | | | |
| --- | --- | --- | --- | --- | --- |
| Subgroup | Best-performing model | All ICU ICC | All ICU CAC | Mega-ROX ICU ICC | Mega-ROX ICU CAC |
| All admissions | Exchangeable | 0.010 | 1.00 | 0.008 | 1.00 |
| All admissions | Block exchangeable | 0.012 | 0.89 | 0.008 | 1.00 |
| All admissions | Discrete time decay | 0.011 | 1.00 | 0.008 | 1.00 |
| Non-elective ICU admissions | Exchangeable | 0.017 | 1.00 | 0.017 | 1.00 |
| Non-elective ICU admissions | Block exchangeable | 0.018 | 0.92 | 0.018 | 0.90 |
| Non-elective ICU admissions | Discrete time decay | 0.017 | 1.00 | 0.017 | 1.00 |
| Invasively ventilated | Exchangeable | 0.020 | 1.00 | 0.020 | 1.00 |
| Invasively ventilated | Block exchangeable | 0.022 | 0.91 | 0.023 | 0.89 |
| Invasively ventilated | Discrete time decay | 0.021 | 1.00 | 0.022 | 0.99 |
| Invasively ventilated during a non-elective admission | Exchangeable | 0.015 | 1.00 | 0.017 | 1.00 |
| Invasively ventilated during a non-elective admission | Block exchangeable | 0.017 | 0.83 | 0.020 | 0.83 |
| Invasively ventilated during a non-elective admission | Discrete time decay | 0.015 | 1.00 | 0.017 | 1.00 |
| Received vasopressors | Exchangeable | 0.011 | 1.00 | 0.011 | 1.00 |
| Received vasopressors | Block exchangeable | 0.013 | 0.86 | 0.013 | 0.86 |
| Received vasopressors | Discrete time decay | 0.012 | 1.00 | 0.012 | 0.99 |
| Received vasopressors during a non-elective ICU admission | Exchangeable | 0.011 | 1.00 | 0.013 | 1.00 |
| Received vasopressors during a non-elective ICU admission | Block exchangeable | 0.013 | 0.84 | 0.015 | 0.85 |
| Received vasopressors during a non-elective ICU admission | Discrete time decay | 0.012 | 1.00 | 0.013 | 0.99 |

Abbreviations: ICC: Intra-cluster correlation coefficient; ICU: intensive care unit; CAC: Cluster auto-correlation coefficient

| **Table S6:** ICCs and CACs for specific subgroups for each of the three within-cluster correlation structures, period length two months | | | | | |
| --- | --- | --- | --- | --- | --- |
| Subgroup | Best-performing model | All ICU ICC | All ICU CAC | Mega-ROX ICU ICC | Mega-ROX ICU CAC |
| All admissions | Exchangeable | 0.010 | 1.00 | 0.008 | 1.00 |
| All admissions | Block exchangeable | 0.011 | 0.90 | 0.009 | 0.88 |
| All admissions | Discrete time decay | 0.011 | 0.99 | 0.009 | 0.99 |
| Non-elective ICU admissions | Exchangeable | 0.017 | 1.00 | 0.017 | 1.00 |
| Non-elective ICU admissions | Block exchangeable | 0.018 | 0.93 | 0.018 | 0.93 |
| Non-elective ICU admissions | Discrete time decay | 0.017 | 1.00 | 0.017 | 1.00 |
| Invasively ventilated | Exchangeable | 0.020 | 1.00 | 0.020 | 1.00 |
| Invasively ventilated | Block exchangeable | 0.021 | 0.92 | 0.022 | 0.90 |
| Invasively ventilated | Discrete time decay | 0.021 | 0.99 | 0.022 | 0.99 |
| Invasively ventilated during a non-elective admission | Exchangeable | 0.015 | 1.00 | 0.017 | 1.00 |
| Invasively ventilated during a non-elective admission | Block exchangeable | 0.016 | 0.88 | 0.018 | 0.88 |
| Invasively ventilated during a non-elective admission | Discrete time decay | 0.015 | 0.99 | 0.017 | 0.99 |
| Received vasopressors | Exchangeable | 0.011 | 1.00 | 0.011 | 1.00 |
| Received vasopressors | Block exchangeable | 0.012 | 0.94 | 0.012 | 0.93 |
| Received vasopressors | Discrete time decay | 0.012 | 0.99 | 0.012 | 0.99 |
| Received vasopressors during a non-elective ICU admission | Exchangeable | 0.011 | 1.00 | 0.013 | 1.00 |
| Received vasopressors during a non-elective ICU admission | Block exchangeable | 0.012 | 0.95 | 0.013 | 0.94 |
| Received vasopressors during a non-elective ICU admission | Discrete time decay | 0.012 | 0.99 | 0.013 | 0.99 |

Abbreviations: ICC: Intra-cluster correlation coefficient; ICU: intensive care unit; CAC: Cluster auto-correlation coefficient

| **Table S7:** ICCs and CACs for specific subgroups for each of the three within-cluster correlation structures, period length three months | | | | | |
| --- | --- | --- | --- | --- | --- |
| Subgroup | Best-performing model | All ICU ICC | All ICU CAC | Mega-ROX ICU ICC | Mega-ROX ICU CAC |
| All admissions | Exchangeable | 0.010 | 1.00 | 0.008 | 1.00 |
| All admissions | Block exchangeable | 0.010 | 1.00 | 0.009 | 0.90 |
| All admissions | Discrete time decay | 0.011 | 0.99 | 0.009 | 0.98 |
| Non-elective ICU admissions | Exchangeable | 0.017 | 1.00 | 0.017 | 1.00 |
| Non-elective ICU admissions | Block exchangeable | 0.018 | 0.93 | 0.018 | 0.94 |
| Non-elective ICU admissions | Discrete time decay | 0.017 | 0.99 | 0.017 | 0.99 |
| Invasively ventilated | Exchangeable | 0.020 | 1.00 | 0.020 | 1.00 |
| Invasively ventilated | Block exchangeable | 0.021 | 0.93 | 0.022 | 0.92 |
| Invasively ventilated | Discrete time decay | 0.021 | 0.99 | 0.022 | 0.98 |
| Invasively ventilated during a non-elective admission | Exchangeable | 0.015 | 1.00 | 0.016 | 1.00 |
| Invasively ventilated during a non-elective admission | Block exchangeable | 0.016 | 0.87 | 0.019 | 0.86 |
| Invasively ventilated during a non-elective admission | Discrete time decay | 0.015 | 0.99 | 0.017 | 0.98 |
| Received vasopressors | Exchangeable | 0.011 | 1.00 | 0.011 | 1.00 |
| Received vasopressors | Block exchangeable | 0.013 | 0.89 | 0.012 | 0.92 |
| Received vasopressors | Discrete time decay | 0.012 | 0.98 | 0.012 | 0.98 |
| Received vasopressors during a non-elective ICU admission | Exchangeable | 0.011 | 1.00 | 0.013 | 1.00 |
| Received vasopressors during a non-elective ICU admission | Block exchangeable | 0.012 | 0.88 | 0.014 | 0.90 |
| Received vasopressors during a non-elective ICU admission | Discrete time decay | 0.012 | 0.98 | 0.013 | 0.98 |

Abbreviations: ICC: Intra-cluster correlation coefficient; ICU: intensive care unit; CAC: Cluster auto-correlation coefficient

| **Table S8:** ICCs and CACs for specific subgroups for each of the three within-cluster correlation structures, period length six months | | | | | |
| --- | --- | --- | --- | --- | --- |
| Subgroup | Best-performing model | All ICU ICC | All ICU CAC | Mega-ROX ICU ICC | Mega-ROX ICU CAC |
| All admissions | Exchangeable | 0.010 | 1.00 | 0.008 | 1.00 |
| All admissions | Block exchangeable | 0.011 | 0.95 | 0.008 | 1.00 |
| All admissions | Discrete time decay | 0.011 | 0.98 | 0.009 | 0.98 |
| Non-elective ICU admissions | Exchangeable | 0.017 | 1.00 | 0.017 | 1.00 |
| Non-elective ICU admissions | Block exchangeable | 0.017 | 0.96 | 0.017 | 0.97 |
| Non-elective ICU admissions | Discrete time decay | 0.017 | 0.99 | 0.017 | 0.99 |
| Invasively ventilated | Exchangeable | 0.020 | 1.00 | 0.020 | 1.00 |
| Invasively ventilated | Block exchangeable | 0.021 | 0.95 | 0.021 | 0.94 |
| Invasively ventilated | Discrete time decay | 0.021 | 0.98 | 0.022 | 0.97 |
| Invasively ventilated during a non-elective admission | Exchangeable | 0.015 | 1.00 | 0.017 | 1.00 |
| Invasively ventilated during a non-elective admission | Block exchangeable | 0.015 | 0.92 | 0.017 | 0.94 |
| Invasively ventilated during a non-elective admission | Discrete time decay | 0.015 | 0.98 | 0.017 | 0.98 |
| Received vasopressors | Exchangeable | 0.011 | 1.00 | 0.011 | 1.00 |
| Received vasopressors | Block exchangeable | 0.012 | 0.93 | 0.012 | 0.95 |
| Received vasopressors | Discrete time decay | 0.012 | 0.97 | 0.012 | 0.97 |
| Received vasopressors during a non-elective ICU admission | Exchangeable | 0.011 | 1.00 | 0.013 | 1.00 |
| Received vasopressors during a non-elective ICU admission | Block exchangeable | 0.012 | 0.92 | 0.013 | 0.94 |
| Received vasopressors during a non-elective ICU admission | Discrete time decay | 0.012 | 0.98 | 0.013 | 0.97 |

Abbreviations: ICC: Intra-cluster correlation coefficient; ICU: intensive care unit; CAC: Cluster auto-correlation coefficient

| **Table S9:** Comparison of ICC estimates for adjusted models with periods of two months. | | | | | | |
| --- | --- | --- | --- | --- | --- | --- |
| Model | Unadjusted | | Adjusted for: rural/regional status, jurisdiction | | Adjusted for: rural/regional status, jurisdiction, age, sex, APACHE2 | |
|  | ICC | CAC | ICC | CAC | ICC | CAC |
| Exchangeable | 0.017 | 1 | 0.017 | 1 | 0.015 | 1 |
| Block exchangeable | 0.018 | 0.88 | 0.018 | 0.88 | 0.018 | 0.79 |
| Discrete time decay | 0.017 | 0.99 | 0.017 | 0.99 | 0.016 | 0.98 |

Abbreviations: APACHE2: Acute Physiology and Chronic Health Evaluation 2; ICC: Intra-cluster correlation coefficient; CAC: Cluster auto-correlation coefficient

**Example sample size calculation**

**OVERVIEW**

This appendix provides a worked example of power and sample size calculation using The Shiny CRT Calculator (<https://clusterrcts.shinyapps.io/rshinyapp/>). This calculator provides a user-friendly interface for researchers to input the data required to generate a sample size calculation for a stepped wedge trial (and for other cluster randomised trial designs). An explanation of the inputs, justifications for selection and how to interpret results presented is provided.

**TRIAL DESIGN**

Our hypothetical trial population for this worked example is patients undergoing unplanned mechanical ventilation at an active trial site. The proposed stepped wedge study design is illustrated in **Figure S1** and is based on:

- 50 ICUs in total
- 11 periods, each lasting two months (i.e. 10 steps).
- An average cluster size for the population of interest for each two month period of 45 (SD 35)
- At each step, 5 ICUs (5 clusters) transition from control (standard care) to intervention.
- At month 22 (period 11), all ICUs have transitioned to the intervention
- The primary outcome is in-hospital mortality during the index hospitalisation within 90 days of ICU admission.

| **Figure S1.** Proposed hypothetical trial design | |
| --- | --- |
| **Period 1:** 50 ICUs allocated to usual care  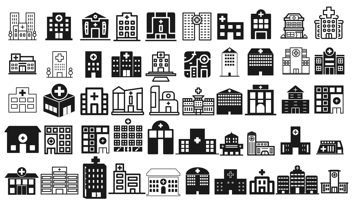 | **Period 2:** 5 ICUs allocated to intervention and 45 ICUs allocated to usual care  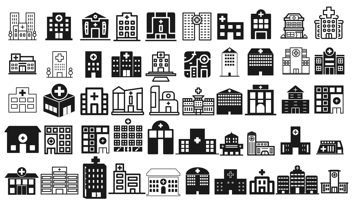 |
|  | |
| **Period 3:** 10 ICUs allocated to intervention and 40 ICUs allocated to usual care  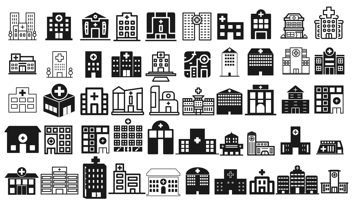 | **Period 4:** 15 ICUs allocated to intervention and 35 ICUs allocated to usual care  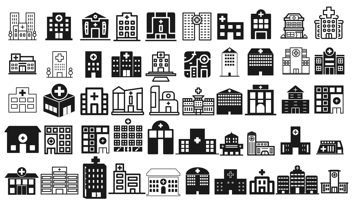 |
|  |  |
| **Period 5:** 20 ICUs allocated to intervention and 30 ICUs allocated to usual care  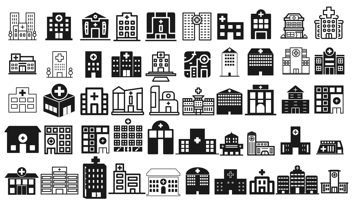 | **Period 6:** 25 ICUs allocated to intervention and 25 ICUs allocated to usual care  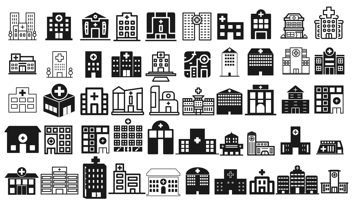 |
|  |  |
| **Period 7:** 30 ICUs allocated to intervention and 20 ICUs allocated to usual care  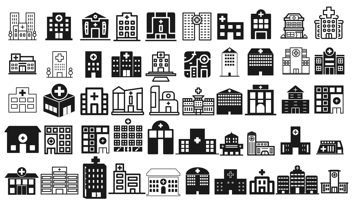 | **Period 8:** 35 ICUs allocated to intervention and 15 ICUs allocated to usual care  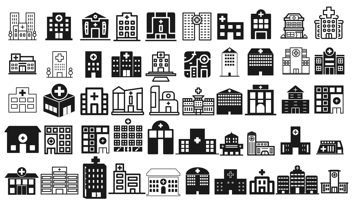 |
|  |  |
| **Period 9:** 40 ICUs allocated to intervention and 10 ICUs allocated to usual care  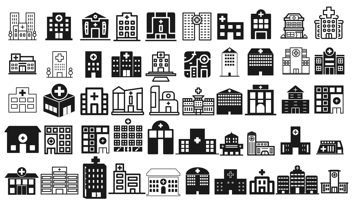 | **Period 10:** 45 ICUs allocated to intervention and 5 ICUs allocated to usual care  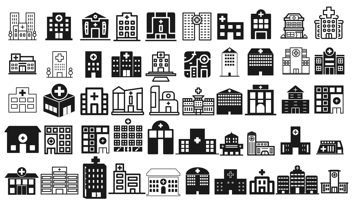 |
|  |  |
| **Period 11:** 50 ICUs allocated to intervention  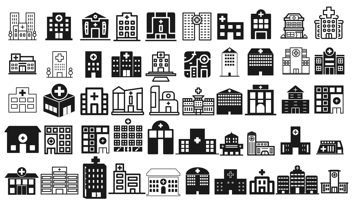 | 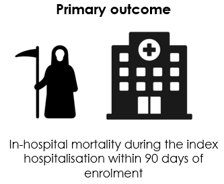 |

**WORKED EXAMPLE (WITH SCREENSHOTS) AND EXPLANATIONS**

**Step 1: Trial Design**

Select ‘**stepped-wedge**’ as the trial design.


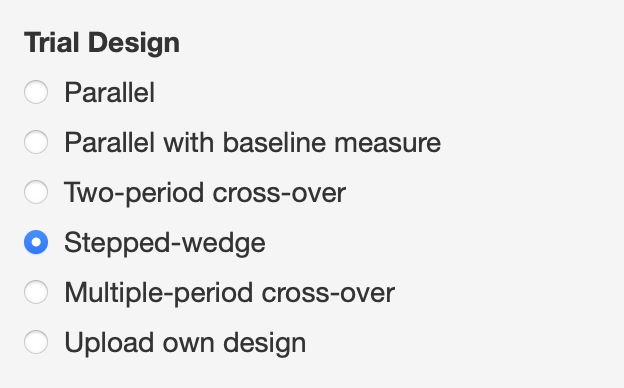


**Step 2: Sampling Structure**

Select ‘cross-sectional sample’. A **cross-sectional sample** is most appropriate for ICU-based studies since a stepped wedge ICU trial will involve sampling different patients at each time point rather than following a single cohort.


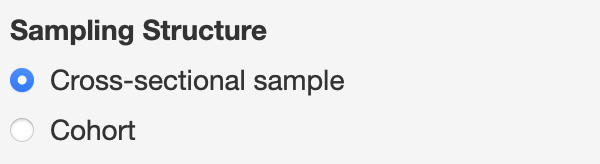


**Step 3: Correlation Structure**

For our trial, the correlation structure that fit the data best based on the Akaike information criterion was the block exchangeable structure (see **Table 4**). This correlation structure is called **two-period decay** in the Shiny calculator.


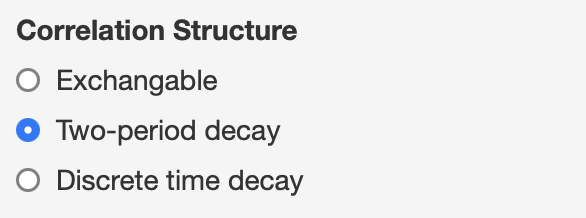


**Step 4: Plot Set-Up**

To understand how the number of clusters at each step affects the trial’s statistical power, begin by selecting the “**Number of clusters vs. Power**” plot. Specify the range of ICUs transitioning per step (sequence) on the X-axis, usually 0-20, since more than 20 ICUs transitioning per step is rarely practical.


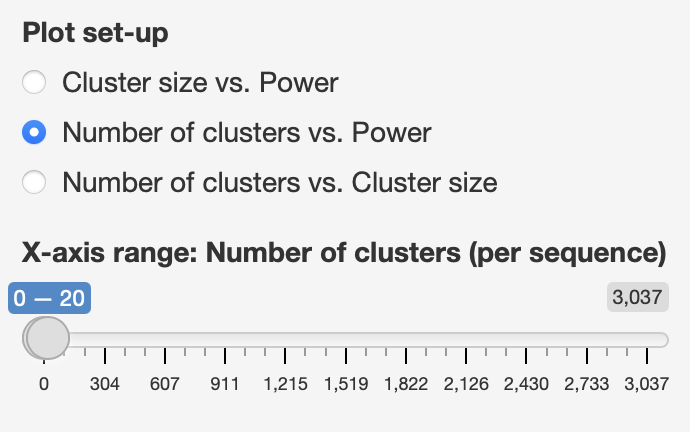


**Step 5: Population of Interest**

The cluster size, number of sequences (steps), and ICC for the population of interest should be entered (see **Table 4** for relevant data for the worked example). The cluster size per period is the mean number of patients in the study population per ICU expected to be admitted in each treatment period. For each 2 month period, the mean number of unplanned ventilated admissions per ICU enrolled in Mega-ROX ICUs in two months is 45 (SD 35). The coefficient of variation of cluster sizes, in this case 0.78, is defined as the ratio of standard deviation of cluster sizes to mean of cluster sizes. Although not relevant to this example, the Shiny CRT calculator cannot combine discrete time decay with variable cluster size. In such circumstances, sensitivity analyses can be conducted using an alternative models and then the most conservative detectable effect used for power calculations. The ICC for this population is 0.018. Since our data provide an exact context-specific ICC for the research and sub-group of interest, we have specified the ICC lower extreme and the ICC upper extreme as 0.018; however, changing these extremes can provide useful information on the sensitivity of calculated power to changes in this parameter.

**
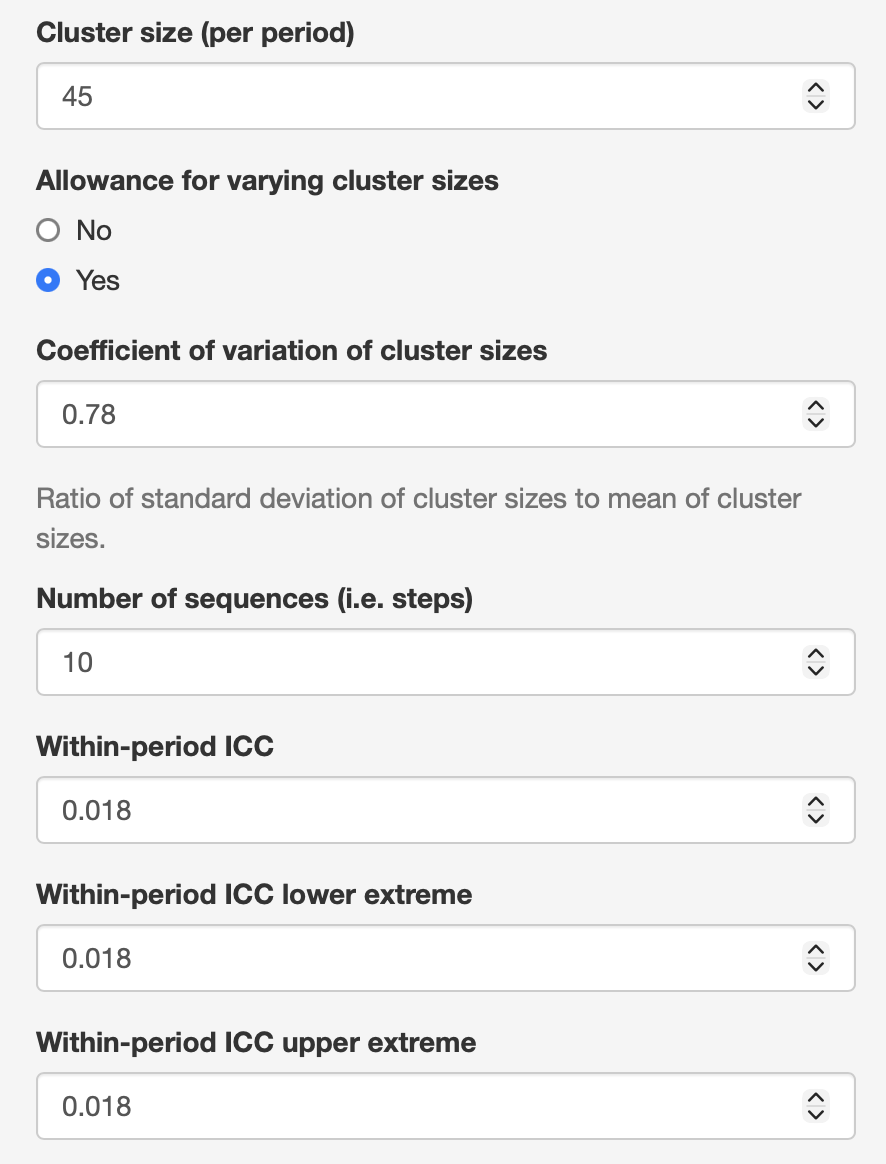
**

**Step 6: Cluster Auto-Correlation**

The cluster auto-correlation for this subset is 0.88 (**Table 4**). An upper (120% of base CAC) and lower (80% of base CAC) measurement is added by the model to aid sensitivity analysis.


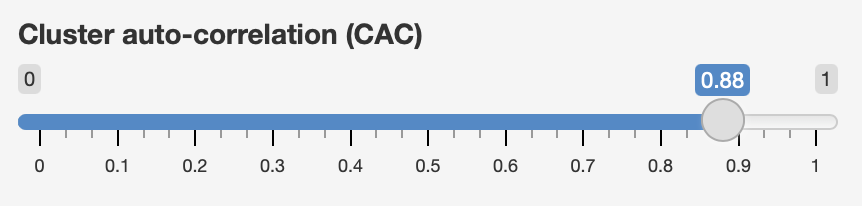


**Step 7: Outcome**

The control event rate (mortality rate in ICU patients) is a binary outcome, so this is selected and the baseline probability of death in the target population (0.23) defines the ‘proportion under control’ (**Table 4**). For our worked example, we have used a nominal effect size of a 2.7 percentage point absolute difference in mortality which mean that the proportion under intervention is set at 0.203. Usually a significance level would be set at 0.05.


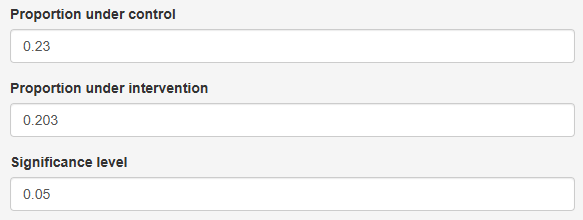


**Step 8: Graphical Interpretation – Power Tab**

The calculator will produce a graphical output as shown in **Figure S2** which shows the increase in power as the number of clusters increases (for a fixed cluster-period size). Hovering the cursor over the curve shows the actual power expected for five ICUs (clusters) per sequence is 81.6%.

| **Figure S2:** Power vs. cluster size for hypothetical trial with parameters shown |
| --- |
| 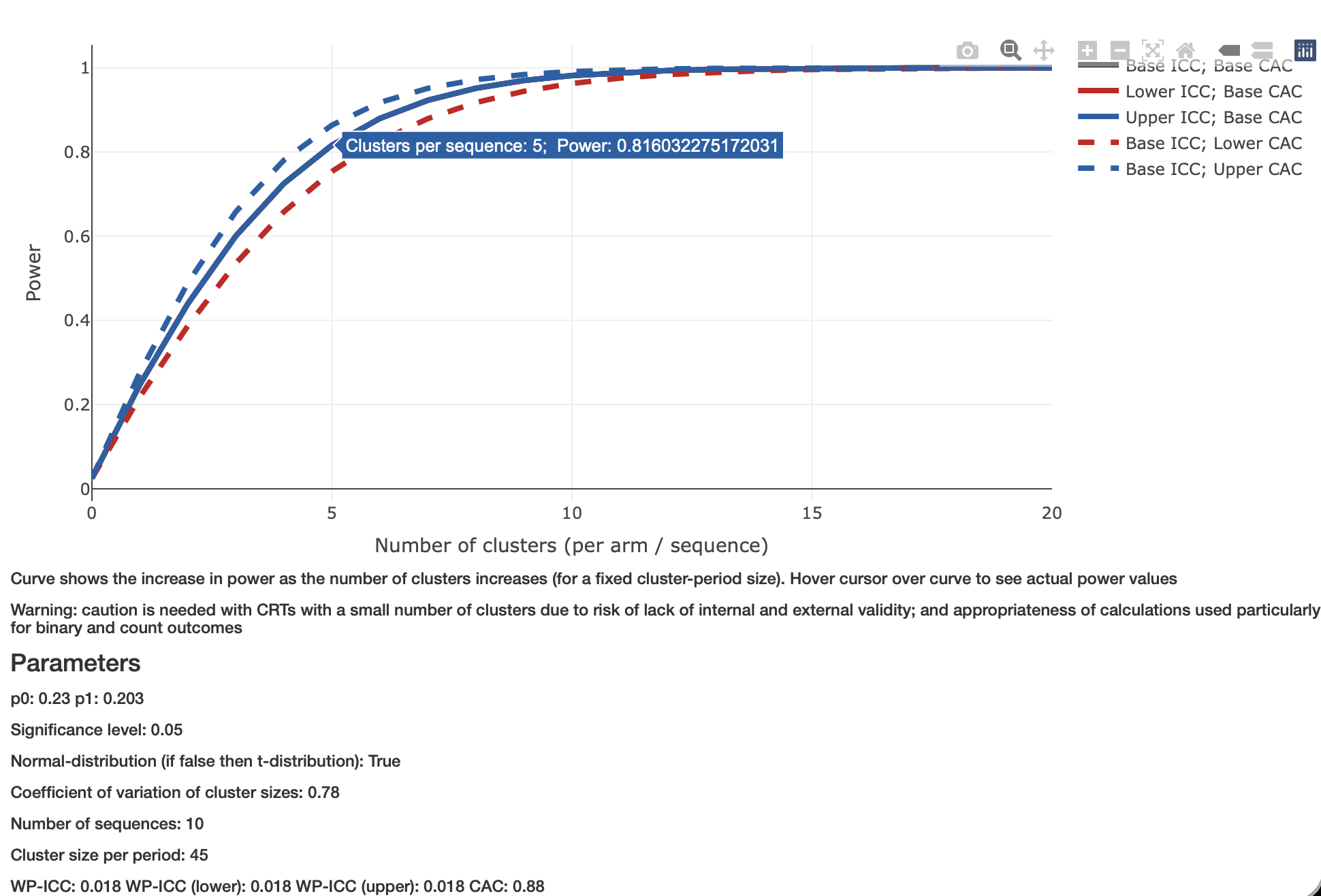 |

**Step 9: Alternative plot set-ups**

Inputting data to provide outputs for number of clusters vs. power and number of clusters vs. cluster size can provide additional useful information in relation to potential trade-offs implicit in alternative trials designs.
